# Exploring performance of athletic individuals: Tying athletic behaviors and big-five personality traits with sports performance

**DOI:** 10.1371/journal.pone.0312850

**Published:** 2024-12-02

**Authors:** Qiong Li, Duo Xiao, Qiong Zeng

**Affiliations:** Jiangxi Gannan Health Vocational College, Ganzhou, Jiangxi, China; Universiti Pertahanan Nasional Malaysia, MALAYSIA

## Abstract

The paper aims to investigate the interplay of sports athletic behavior between big-five personality traits and sports performance in China. The study acquired cross-sectional responses on the questionnaire from 260 Chinese sports athletes, including males and females. Fuzzy analysis techniques were applied to examine the results of the study. Fuzzy TODIM assessment, and fuzzy decision analysis technique applied to draw inferences. The results established that big-five personality traits are significant predictors of athletic behaviors; however, neuroticism is found to be insignificant. The interplay of athletic behavior of sports athletes is also significant in sports game performance. The role of athletic behavior in the nexus between personality traits and sports performance is also significant. Gender also has a significant role in behavior and sports performance. Chinese educational institutional and sports administrations should strive to encourage psychological training programs are enhance mental resilience for effective performance. Valuing sports opportunities for females to shape gender equality would promote sports performance at the school and college levels. Therefore, policy makers need to take such initiatives for a physically academic grooming of the individuals.

## 1. Introduction

There has been a meteoric rise in the number of athletes hailing from Ganzhou city of Jiangxi province in China renowned for its innovation and fast urbanization [1]. Around 1.2 million athletes, both amateur and professional, are expected to be participating in the city’s sports scene in 2023. This is a significant increase from the 800,000 athletes reported in 2018, suggesting a gain of 50% over the last five years. A greater emphasis on physical and fitness, together with rising expenditures on sports facilities, has contributed to this upsurge in participation in athletic events and other forms of physical activity. The Ganzhou city of Jiangxi province needs to improve its sports facilities and ancillary services if this upward trend is to persist. Due to the increasing number of athletes, the city’s current inventory of 500 sports venues—including stadiums, gyms, and community sports centers—is quickly becoming inadequate. In order to provide athletes with the necessary areas for training and competition, an extra 300 sports facilities are projected to be required to fulfill the demand appropriately. Even more specialized facilities are desperately needed; for example, only around 15% of athletes have access to high-performance training centers at the moment. Athletes looking to improve their performance in competition would greatly benefit from increasing this availability to at least 40% [2]. At now, there are around 2,500 licensed coaches in Ganzhou city of Jiangxi province. However, in order to ensure that each athlete receives individualized and effective training, it is projected that at least 4,000 coaches will be required.

The intricate network of relationships between athletic behaviors and game performance has recently come to the forefront of sports psychology research [3]. These characteristics heavily influence athletes’ conduct and results, the most important of which are the Big Five: openness, Conscientiousness, extraversion, agreeableness, and neuroticism [4]. Understanding these dynamics is crucial for China, a country with a growing influence in international sports, to maximize the growth and performance of its athletes [5]. There is a noticeable lack of research on how the Big Five personality qualities affect Chinese athletes’ conduct and performance, even though sports science has come a long way. This highlights a gap in both academic study and real-world application. Researching the relationship between character traits and athletic success in China is fraught with difficulties due to the country’s rich cultural, social, and historical background [6]. It means that different facets of personalities may appear in Chinese athletes non-consciously since they have grown within the framework of Confucianism which entails collectivism, hierarchy, and systematic harmony. The concept of humor and endurance to restraints may also work differently in a society that greatly appreciates extraversion, which is one of the personality variables often described in terms of friendliness and assertiveness. Speaking of Chinese values, the perception of which is crucial to convey the idea of the necessary endeavor, hard work, and persistence, it is aligned with the trait of Conscientiousness, which is most often associated with the characteristics above [7]. This type of current research, however, is primarily rooted in the Western context and, to the authors’ knowledge, has not undertaken an extensive analysis of cultural complexities within this area of research. Therefore, the results, which include factors regarding the higher the level of muscularity, the more muscular negatively predicts body image, the more positively muscularity relates to satisfaction with appearance, and the lower the BMI, the more positive the body image, might not apply to Chinese athletes.

Moreover, it could be suggested that Chinese athletes can respond to some constructs of personality with specific behavioral and performance outcomes due to the interaction of personality-related variables with the specific experiences that are typical for athletes who have to face numerous strict requirements, including strict training schedules, high personal expectations, and early sport specialization. For instance, it can be assumed that due to the Chinese sports system that restricts athletes’ freedom in many ways, athletes who score high in neuroticism would be even more anxious and stressed out, which would negatively influence their performance. Specifically, it can be proposed that persons who have a higher Conscientiousness score and a lower Neuroticism score are able to cope with these kinds of stresses better because they are more stable and stronger. From these encounters, it is possible that the understanding of the Big Five personality characteristics in the Chinese sports environment might enhance the coaching tactics, psychological support, and methods of managing athletes. Studies conducted recently often use frameworks and assessment instruments derived from the West, which could not adequately account for the cultural nuances of Chinese athletes. The lack of study in this area makes it harder to create theories and models that are applicable to Chinese culture and restricts the use of psychological insights to improve Chinese athletes’ performance [8]. Filling this void is essential for sports psychology in China to progress in both theory and practice; however, the research objectives are as follows,

1. *To examine the influence of personality traits on athletic behavior of sports athletes*.
2. *To examine the influence of personality traits on sports performance of sports athletes*.
3. *To examine the influence of athletic behavior on sports performance of sports athletes*.
4. *To examine the athletic behavior mediating influence between personality traits and sports performance*.

The contribution of research covers many theoretical and practical aspects. Athletes’ actions and results on the field may be better understood by looking at how the Big Five personality qualities (Openness, Conscientiousness, Extraversion, Agreeableness, and Neuroticism) play out. Incorporating findings from personality psychology into sports psychology theoretically increases the current corpus of knowledge in the field. It reveals that motivation, stress tolerance, collaboration, and leadership are constants that are influenced by one’s character traits. In extending the application of the so-called Big Five model, that is, a set of personal features now characteristic of the field of general psychology into the sphere of athletic performance, the value of the above characteristics within the sports field is additionally broadened. Hence, a more exhaustive account of the identity of the personality-performance relationship that is relevant in competitive settings may be realized with this multidisciplinary synthesis. The findings of the study have significant implications for athlete nurturing, coaching strategies, and increase in performance. Coaches might adjust training schedules according to the psychological characteristics of performers because some aspects influence athletic activities. Athletes who are open may find creative and diversified training regimens by doing well, while athletes with high conscientiousness may fare better under strict and structured training regimens. Improving the mental state and coping mechanisms may be gained through the creation of psychological treatments targeting anxiety and stress, which will depend on personality traits like neuroticism. The findings of this study have implications for team building and recruitment efforts since they pinpoint individual characteristics that are associated with productive leadership and collaboration.

Personality study is important for enhancing team and individual performance in sports because it provides both theoretical understanding and practical information. The second part explains the review of the literature. The third part elaborates on the method of inquiry. The fourth and fifth part explains the results and discussion. The last section presents the conclusion and implications.

## 2. Literature review

### 2.1 Athletic performance

Physiological and psychological elements, as well as environmental and technological influences, are all part of what makes an athlete perform well in sports [9]. Acquiring this knowledge is crucial for peak performance, injury prevention, and athletic greatness. This article delves into important factors that impact sports performance, such as training and diet, mental factors, and technological developments [10]. Athlete physiological traits are the bedrock of success in athletics. Strength training, cardiovascular fitness, flexibility, and body composition are all part of this. To exert force and keep going for a long time, you need strong muscles [11]. Increased mobility, or flexibility, lessens the likelihood of injury and boosts efficiency. The lean-to-fat mass ratio, in particular, influences speed, power, and agility [12]. Specific training regimens that include aerobic conditioning, flexibility exercises, and weight training may enhance these physiological traits [13].

In order to improve certain aspects of sports performance, training regimens are meticulously crafted [14]. A fundamental principle in sports training is periodization, which entails changing the intensity and amount of exercise at predetermined intervals. With this method, you may avoid overtraining and perform at your best when it matters most. A well-rounded training program must include strength training, cardio, plyometrics, and exercises tailored to the individual sport [15]. In order to sustain and enhance the benefits of exercise, proper nutrition is essential. Essential for energy generation, muscle repair, and general physical are macronutrients (carbs, proteins, and fats) and micronutrients (vitamins and minerals). During intense physical activity, carbohydrates are the fuel of choice, while proteins help with muscle repair and development [16]. Even though they don’t get much attention, fats are crucial for activities that last a long time. Another important consideration is staying hydrated since even a little loss of fluids might hinder performance [17]. If you want to perform at your best, you need a nutrition plan that works with your training intensity and your objectives.

There is a strong correlation between psychological aspects and performance in athletics. Elite athletes are defined by a set of psychological characteristics that include mental toughness, drive, concentration, and the ability to handle stress [18]. Athletes who possess mental toughness are able to push through difficult practices and games. Athletes are driven to create and accomplish objectives by motivation, whether it’s internal or external. Competitors must be able to focus or keep their attention on the task at hand, even while they are under time constraints. Improper management of stress and anxiety may impair performance. To improve these mental qualities, people commonly utilize techniques like mindfulness training, visualizing a successful outcome, and goal-setting. Athletes greatly benefit from the guidance of sports psychologists, who can teach them coping mechanisms and mental toughness [19]. Technological improvements have transformed sports performance. Fitness trackers, smartwatches, and other wearable tech monitor vitals, including heart rate, sleep duration, and activity levels, in real-time. Performance, recuperation, and general physical may be tracked using this data, which is useful for coaches and players alike. With the use of video analysis technologies, athletes can see their technique in great detail, which allows them to make fine-tuned tweaks and progress [20].

Athletes are required to learn about dealing with the unavoidable risk of injury if they want to keep performing at a high level. The use of strength training, flexibility exercises, and proper warm-up and cool-down protocols may lessen injuries. Injuries must be treated and diagnosed promptly to avoid long-term consequences [21]. A full recovery is possible with the help of rehabilitation programs that include strength training, physical therapy, and a slow but steady increase in activity levels. Athletes rely on physiotherapists and sports medicine experts to help them stay physically and perform at their best [22]. Athletes’ innate abilities are also impacted by their genes. Varieties in heredity have the potential to influence not just physical attributes like heart rate and muscular fiber composition but also mental qualities like drive and concentration. Training and contextual variables considerably alter real performance, while hereditary factors provide a foundation for potential. Achieving athletic greatness is a challenging endeavor, made more so by the interaction between genetics and training. There is a constant feedback loop between an athlete’s physiological, psychological, environmental, and technical elements that determine their performance on the field [15]. A comprehensive strategy that incorporates specific exercise, physically eating, mental toughness, and cutting-edge equipment is necessary to achieve peak performance. Athletes may realize their dreams, become the best they can be, and win more often if they take the time to learn about and use these factors. Consequently, our ability to fine-tune athletic performance and perhaps extend the limit of human physiology will stand to be advanced by leaps and bounds due to the knowledge that has been gained and is bound to continue being revealed from the growing sub-domain of sports science [14].

### 2.2 Personality traits and athletic performance

The studies that compare personality characteristics and athletic performance have received a considerable amount of attention in both the fields of psychology and sports science [23]. Each person’s unique personality and how it may affect their performance on the field may be better understood by looking at the Big Five personality traits. The major ones include the Big Five personality tests that comprise Openness to Experience, Conscientiousness, Extraversion, Agreeableness, and Neuroticism [24]. This entails ideas and getting lost in them, marveling, and the propensity to accept new types of attributes linked to an individual’s Openness to Experience. As for training, it has to be noted that athletes are characterized by higher results on the Openness to Experience scale, which is why they can be more creative and flexible. This makes them new to most things, and since they excel in training, they are open to new things [25]. Based on the existing scholarly literature, athletes with this trait are likely to be more resilient to the volatility characteristic of this industry [26]. That is what it means to be conscientious: self-control, structuring, and direction. Regarding training, it has been established that high Conscientiousness is manifested in athletes by their ability to be dependable as well as thorough. Their dedication to their objectives is evident in the way they follow rigorous training regimens and exhibit a great level of self-control. Because conscientious athletes are better able to concentrate for lengthy amounts of time, manage their time wisely, and keep themselves motivated, research has shown that Conscientiousness positively correlates with athletic performance [27].

Exuberant, gregarious, and outgoing conduct is the hallmark of extraversion. Typically, outgoing athletes are full of energy, confidence, and action [2]. They do best in group settings and often outperform their teammates in team sports that emphasize talking to one another. In general, these athletes are more active and inclined to take part in athletic competitions. According to studies, competitive environments may be quite motivating for extroverted athletes, who may find that they perform even better [3]. A greater link is shown in team sports compared to individual sports, suggesting that the impact of extraversion on performance may differ across different types of sports [1]. Characteristics like a desire to avoid confrontation, empathy, and collaboration are reflected [4]. When it comes to sports, athletes who score high on the Agreeableness scale tend to be great team members. Team sports, which rely heavily on cooperation and mutual aid, are an ideal environment for these traits to flourish. When athletes are pleasant to be around, it helps create a good vibe on the team and makes everyone feel welcome [6]. Agreeableness improves team cohesiveness and performance as a whole, but it has no clear correlation with individual performance metrics [8].

Characteristics of neuroticism include irritability, impulsivity, and anxiety. Athletes with a high level of neuroticism may struggle to control their emotions and stress, which in turn impacts their performance [11]. Low self-confidence, inability to concentrate, and erratic emotional responses are all symptoms of high anxiety and emotional volatility. Although anxiety is a negative state, there is information concerning the beneficial effects of anxiety, namely drive and increased consciousness. The technique is to channel worry in a positive direction in order to prod yourself into action without being consumed by it. Naturally, the athletes who are lower on the neuroticism scale are calmer and more resistant to stress, which is proven by the data, according to which they perform better in high-stress situations [10]. From the previous findings [7, 12], it is evident that the five basic dimensions of personality significantly affect the performance of an athlete in terms of the type and profundity though varying depending on the sport and the surrounding condition [12]. Especially in the activities that require commitment, planning and determination in the long run, Conscientiousness appears as the most reliable and stable predictor of the performance.

In contrast to individual sports, the level of extraversion and agreeableness is higher in team sports due to the nature of the relationship between players in any team. Neuroticism is advantageous to athletes by helping to avoid emotional overreaction and anxiety during pressure and stress. At the same time, Openness to Experience might be beneficial to athletes in that it leads them to be creative in solving problems in the field.

### 2.3 Big five personality traits and athletes’ behavior

Nowadays, athletes’ personality traits and their impact on performance are one of the most explored categories in psychology and sports science [13]. An in-depth model for understanding individual variations and their implications on athletes’ behavior may be found in the Big Five personality traits: Personality dimensions such as Open to Experience, Conscientious, Extraverted, Agreeable, and Neuroticism [14]. The person is always ready to experiment; they are inventive and eager to discover something new. As an outcome, they may be innovative in dealing with their sport as an implication of exploring new actions. Such individuals with perceive and remember what to do when there are unpredictable challenges or when the conditions change frequently [15]. As the compiled research revealed, such an athlete performs optimally in a sport when it involves a high level of speed, and decision-making skills where one needs to think outside the box [21]. The meaning of conscientiousness is being able to display self-control, organization, and purpose. What sort of work schedules characterize their training regimens? The results show that athletes with high Conscientiousness scores are highly reliable and are imbued with strong work ethics. They have short-term objectives, always plan for the activities in advance, and are persistent in the event that they face challenges [28].

The sports athletes usually have a system in place and stick to it, which allows them to train and perform consistently [22]. Research shows that Conscientiousness is a significant predictor of athletic performance. This is because conscientious athletes are able to stay motivated and focused for extended periods, which allows them to constantly develop and accomplish more [26]. Being extroverted means being lively, friendly, and outgoing. Team sports, which rely heavily on communication and interaction, are ideal for extroverted athletes because of their comfort in social situations [24]. Both in training and competition, these athletes are full of energy and confidence. Their sociable personality may improve team relations, creating an atmosphere where everyone works well together [27]. According to studies, extroverted athletes have an advantage in sports since they are more prone to exercise and like the competitive nature of the activity [25]. Whether a sport places more value on individual effort or teamwork determines the extent to which extraversion plays a role.

When it comes to sports, athletes who score high on the Agreeableness scale tend to be the most trustworthy and cooperative teammates [19]. These traits really shine in team sports when helping one other out and working together are the keys to victory. Players who are easy to get along with tend to boost morale and productivity on the field [22]. Agreeableness may not have a clear correlation to individual performance indicators, but it does improve team cohesiveness and, by extension, collective results [20]. Characteristics of neuroticism include irritability, impulsivity, and anxiety. Athletes with high levels of neuroticism may have trouble controlling their emotions and dealing with stress, which shows in their performance [15]. When people are anxious and emotionally unstable, they may find it difficult to concentrate, make bad decisions, and falter under pressure. According to research, may actually boost performance by making people more motivated and attentive [14]. Athletes who score high on the neuroticism scale must learn to control their emotions via the use of physically coping mechanisms. Because they are able to maintain their composure and concentration under intense scrutiny, athletes who score lower on the neuroticism scale tend to do better [9]. The Big Five personality characteristics have a substantial effect on athletes’ actions, according to a meta-analysis of research; the extent to which this effect varies by sport and environment [16]. Particularly in sports that call for sustained effort and careful preparation, Conscientiousness has shown to be an important characteristic associated with peak performance [17]. Team sports, which rely heavily on communication and cooperation among players, are ideal for those who are extroverted and agreeable. Low degrees of neuroticism aid athletes in keeping their cool and concentrating under pressure, while openness to experience may improve behavior by encouraging innovation and adaptation. Correspondingly, It is hypothesized that;

*H1*: *Personality traits are significant with athletic behavior of sports athletes*.
*H2*: *Personality traits are significant with sports performance of sports athletes*.
*H3*: *Athletic behavior is significant with sports performance of sports athletes*.
*H4*: *Athletic behavior mediates between personality traits and sports performance of sports athletes*.

## 3. Methodology

### 3.1 Population and sample

**Fig 1:**
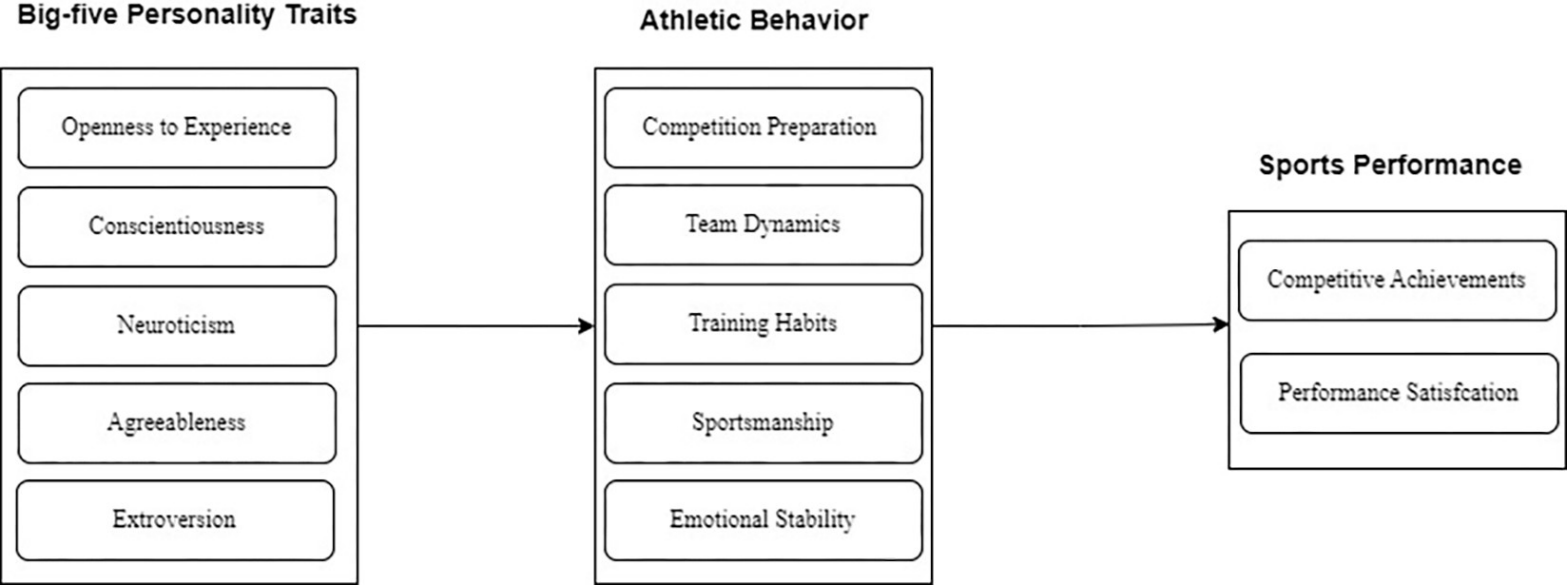
Research structure.

Fig 1 shows the research model of the study. Participants in this research are athletes based in the Chinese city of Ganzhou city of Jiangxi province. Basketball, soccer, swimming, and track and field are just a few of the many sports practiced by the city’s diverse athletic population in Ganzhou city of Jiangxi province, which has become famous for its fast expansion. The athletes included in this research span a broad variety of levels of competition and sports, from amateurs to professionals. Because of this variety, we have a great chance to study the connections between the Big Five personality characteristics and athletic success and behavior. We used cluster sampling to guarantee a cross-section of athletes and skill levels. Athletes ranging in age from 19 to 35 made up our sample. However, the sample of the study includes Chinese sports athletes in Ganzhou city of Jiangxi province China. Notably, none of the minor age athlete is selected for this study.

We made sure there was a balanced representation of men and females by including. The population of the study was unknown. However, clusters were made to trace the sample accordingly. The 300 questionnaires were distributed among the respondents for response acquisition. Males and female were collectively approached. From 300 potential respondents, 280 responses were received back, and 20 individuals didn’t want to participate in our research. However, these were excluded. From 280 responses, 15 responses were incomplete and inappropriately responded. These incomplete responses are also excluded to control estimation issues at the next stages. Hence, the actual respondent size of the study is 265 sports athletes. To ensure relevance, accuracy, and consistency, the study reached out to several Ganzhou city of Jiangxi province-based sports groups, colleges, and professional teams to enlist their participants. In order to be considered, athletes needed to have a minimum of two years of competitive experience and to have participated at a regional or national level. The recruitment period of the sample is tabulated in Table 1.

**Table 1:** Recruitment period of sample and research activity schedule.

*Activity*	*Date(s)*	*Status*
Study Starting Date	25-1-2023	Completed
Documents Submission for Ethical Approval	1-4-2024	Completed
Ethical Approval Obtained	14-4-2024	Completed
Recruitment Period of Sample Started	15-4-2024	Completed
Recruitment Period of Sample Closed	15-5-2024	Completed
Pre-analysis activity Started	16-5-2024	Completed
Analysis Completed on	23-5-2024	Completed
Research Paper Completed	18-6-2024	Completed
Study Closing Date	1-7-2024	Completed

### 3.2 Sampling technique–Cluster sampling

**Fig 2:**
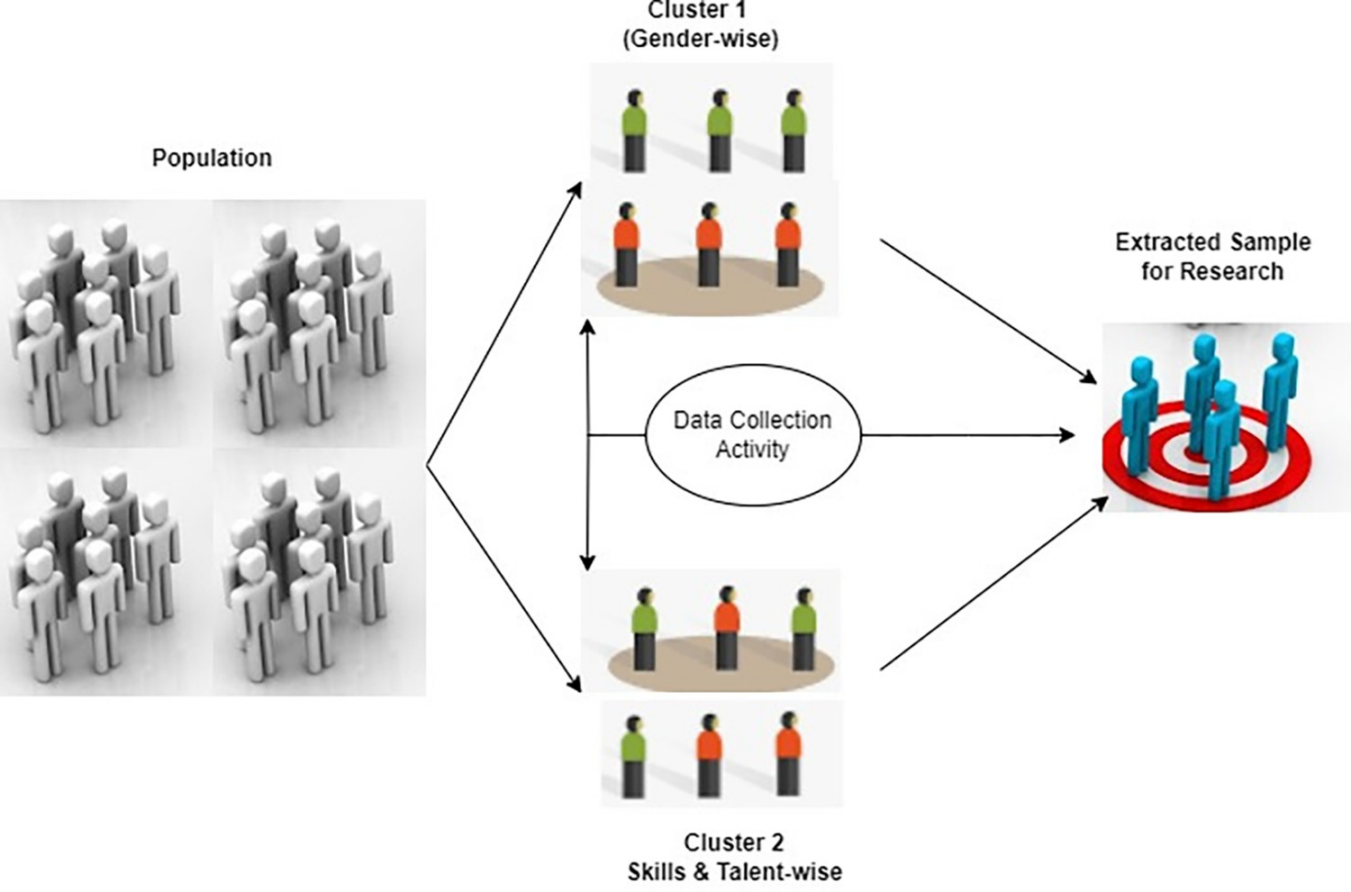
Cluster sampling technique to trace the sample of study.

Using a cluster sampling approach (Fig 2), we collected data on athletes’ performance, behaviors, and Big Five features in Ganzhou city of Jiangxi province, China. A wide range of athletic abilities and specializations were represented by the athletes hailing from Ganzhou city of Jiangxi province, a dynamic metropolis known for its vibrant sports culture. Since sports clubs, colleges, and professional teams are the major gathering places for city athletes, we located them in order to conduct cluster sampling. To ensure variety and representativeness, we randomly selected some sub-clusters from each major cluster. For example, in this group of schools, we considered those schools that were very strong in sports especially. Thirdly, to ensure the study’s diversity, we selected teams within the professional sports cluster but from different games. The given method could be applied to collect data from several types of athletes from track and field, swimming, soccer, and basketball, among others. We contacted the athletes from each of the sub-cluster. We invited them to participate in the research by getting in touch with individual sports organizations and teams after each sub-cluster selection. This method ensured that we had a normal sample population that closely reflected other athletic people of Ganzhou city of Jiangxi province. Our results are more general and give a comprehensive overview of Ganzhou city of Jiangxi province’s sports community because, collecting information through cluster sampling, we surveyed a large number of athletes quickly.

### 3.3 Measurement of variables

In evaluating the participants’ character attributes, we employed [29] the Big Five Inventory (BFI), a well-known psychological tool to assess the five essential aspects of personality. This assessment or personality measures such aspects as neuroticism, agreeableness, openness to the experience, extraversion, conscientiousness, and the second aspect of conscientiousness on the Likert scale, which ranges between 1(totally disagree) and 5 (totally agree).

[30] presented the initial scales to measure athletic behavior. Later, [31] used it. Following this, the current study used similar questionnaire to assess athletic behavior. The study used previously available scale. Training routines approach to competition preparation, team chemistry, and values about sportsmanship were among the many areas that were evaluated. Athletes’ training habits and competitive mindsets were assessed by collecting responses on a Likert scale, which measures the frequency and intensity of certain behaviors.

Following [32] our study assesses the athletic performance on the reported questionnaire. To assess the athletic improvement, participants documented their personal best in the various athletic activities they undertook through the assessment period on strength, endurance, and speed. They also described achievements accomplished in regional and national competitions. Finally, to complete the evaluation of athletic accomplishments, players reflected on how content they felt with their performance and areas that stand out for enhancement. Questionnaire assessments promoting the identification of the correlation between athletes’ personality traits and their athletic behavior and outcomes in Ganzhou city of Jiangxi province, China, involved conducting a detailed analysis of the relationship between different personality characteristics, athletes’ behaviors, and their performance.

### 3.4 Ethical approvals

The Institutional Review Board (IRB) and the Ethical Compliance Research Board (ECRB) of our institution approved this work (*attached in supplementary materials*). To ensure all the parts of the study methodology met the highest standards of ethicality. The study defended the rights as well as the safety of the participants; the study methodology was scrutinized. Each participant produced their consent which showed that their involvement in the study was purely on a voluntary basis (*attached in supplementary materials*).

### 3.5 Estimation technique

The Best-Worst Method (BWM) is known for giving more reliable results because of its structured pairwise comparison method. This is one of the main reasons it was chosen for this study. Also, decision-makers think BWM is easy to understand and fits well with how they normally make decisions. These are the steps that need to be taken:

**Step 1:** Figure out the factors for making a decision: First, we list factors (c1, c2,…, cn) necessary for making a choice.
**Step 2:** Choose the best and worst criteria: The people making the decisions list the most desired (best) and least desired (worst) factors without directly comparing them.
**Step 3:** Setting values for preferences: People making decisions rate the factors on a scale from 1 to 9 to show which one they think is the best compared to the others. By going through this process, a Best-to-Others vector is made, which shows how important each factor is compared to the others.

$$A_{B}=(a_{B1},a_{B2},\ldots ,a_{Bn})$$ (1)

For our purposes, let’s say that aBj is the best criterion B’s choice over criterion j. Notably, it’s clear that aBB = 1.

**Step 4**: We use numbers from 1 to 9 to figure out which criterion all other criteria are more likely to be chosen over. After this process, the Others-to-Worst vector is created. In this vector, ajW is the choice of criterion j over the worst criterion W. Of course, aWW equals 1.

**Step 5:** Finally, we find the best weights (w*1, w*2,…, w*n). These ideal weights make the criteria more evenly distributed, guaranteeing a thorough and fair evaluation.


$$\begin{array}{c} minmax\;\{\left|\frac{W_{B}}{W_{j}}-abj|,|\frac{W_{j}}{W_{w}}-ajw\right|\}\\ S.t\\ \sum_{j}\;w_{j}=1\\ w_{j}\geq 0,for\;all\;j \end{array}$$ (2)

By answering this problem, we find the most useful weights mentioned in Eq (3) and onwards.


$$\begin{array}{c} min\xi \\ s.t.\\ |w_{B}-a_{Bj}w_{j}|\leq \xi ,for\;all\;j\\ |w_{j}-a_{jw}w_{w}|\leq \xi ,for\;all\;j\\ \sum_{j}\;w_{j}=1\\ w_{j}\geq 0,for\;all\;j \end{array}$$ (3)

The TODIM method is different from other Multiple Criteria Decision Making (MCDM) methods like AHP and TOPSIS because it doesn’t give each option a final grade or score but instead looks at how dominant each one is overall. The TODIM method is designed to work with MCDM situations where factors are set as clear numbers. To deal with the problems that come up with MCDM in unclear settings, the TODIM method has been changed to work in fuzzy domains, as shown by current fuzzy TODIM models. These changes aim to lessen the effect of decision-makers’ biases and beliefs on other ratings. Language variables, shown by triangle fuzzy numbers, express attribute values. Using the TODIM framework, these fuzzy numbers are used to determine the pros and cons of each option compared to the others, as well as their total value and control degree. The options are finally ranked based on these final general numbers (Table 2).

**Table 2:** Fuzzy weights of the parameters.

Parameters	Weights	Parameters	Weights
Openness to Experience	0.843	Team Dynamics	0.602
Conscientiousness	0.648	Sportsmanship	0.732
Extroversion	0.863	Training Habit	0.536
Agreeableness	0.454	Emotional Stability	0.615
Neuroticism	0.751	Competitive Achievement	0.924
Competition Preparation	0.272	Performance Satisfaction	0.918

**Step 1:** Explain what linguistic variables and fuzzy numbers are. A linguistic variable is a set of values that are represented in language. Set S is a pre-defined collection of linguistic terms {sf | f = 0, 1,…, T}, where sf is the fth linguistic variable in S and T+1 is the number of elements in S.


$$\mu_{\hat{d}}(x)=\left\{ \begin{array}{cc} 0, & x<d^{l},\\ \frac{\left(x-d^{l}\right)}{\left(d^{m}-d^{l}\right)}, & d^{l}\leq x\leq d^{m},\\ \frac{\left(d^{u}-x\right)}{(d^{u}-d^{m}),} & d^{m}\leq x\leq d^{u},\\ 0, & x>d^{u}, \end{array}\right.$$ (4)

**Step 2:** Figure out the gains and losses. To find out what each option gains and loses compared to the others, we need to compare the trait numbers pair by pair. Let `xij and `xkj represent the property values of options Ai and Ak, respectively. To make performance variations between attributes more consistent, we use the attribute with the most weight as the reference attribute for these calculations, considering each attribute is important.


$$d({\hat{x}}_{ij},{\hat{x}}_{kj})=\sqrt{\frac{1}{3}[{\left(x_{ij}^{l}-x_{kj}^{l}\right)}^{2}+{\left(x_{ij}^{m}-x_{kj}^{m}\right)}^{2}+{\left(x_{ij}^{u}-x_{kj}^{u}\right)}^{2}]}$$ (5)

**Step 3:** Figure out how much each criterion is worth concerning the others (Cj).

To use the TODIM method, you must project the differences between any two possible outcomes onto a reference property.

## 4. Results & findings

### 4.1 Fuzzy TODIM assessment findings

There are a number of important takeaways from studying the impact of the Big Five personality qualities on Chinese athletes’ performance: agreeableness, neuroticism, openness to experience, extraversion, and Conscientiousness. Because of the importance of motivation, coping mechanisms, collaboration, and general mental resilience in high-pressure athletic settings, these characteristics greatly contribute to players’ achievements. Athletes’ capacity to adapt and innovate during training and competition is enhanced when they exhibit openness to experience, which is characterized by a desire to participate in new experiences and creative thinking. In order to stay ahead of the competition, athletes who score high on the openness to new experiences and ideas dimension are able to adapt to a wide range of situations. Quick thinking and the capacity to apply new ideas may contribute to greater performance in dynamic sports environments. Therefore, adaptability is especially useful in such situations (Table 3).

**Table 3:** Estimating $A_{B},\;w_{j},\;\mu_{\hat{d}}(x)$, $G_{ik}^{j}$ fuzzy matrix score.

Fuzzy Hierarchy	*A*_*B*_	*w*_*j*_	$\mu_{\hat{d}}(x)$	$G_{ik}^{j}$	*w*_*jr*_
C11	(1,3,3)	(1,1,5)	(1,2,6)	(1,1,3)	(1,7,4)
C12	(3,1,2)	(1,9,9)	(1,3,8)	(1,3,7)	(1,1,9)
C13	(1,2,2)	(1,3,9)	(1,1,9)	(1,4,1)	(1,3,4)
C21	(1,1,6)	(4,1,4)	(1,2,6)	(1,2,4)	(1,8,3)
C22	(1,2,8)	(1,2,7)	(1,1,5)	(1,9,2)	(1,2,4)
C23	(1,2,7)	(1,7,3)	(2,1,5)	(1,8,8)	(1,2,4)
C31	(1,1,9)	(1,7,1)	(1,2,5)	(1,6,6)	(1,3,1)
C32	(2,2,1)	(2,2,7)	(2,4,6)	(1,6,5)	(1,2,4)
C33	(1,2,8)	(4,2,5)	(1,2,4)	(2,3,5)	(3,9,6)
**C1**	(1,5,7)	(1,1,1)	(1,1,7)	(1,2,6)	(9,1,5)
**C2**	(1,3,3)	(1,1,3)	(1,1,6)	(1,8,6)	(5,3,3)
**C3**	(1,4,3)	(3,3,4)	(1,2,1)	(2,5,3)	(5,5,3)
Fuzzy Mean-Variance	0.272	0.11	0.244	0.148	0.179
Fuzzy R-Sq	0.638	0.779	0.879	0.781	0.693

Improved athletic performance is significantly linked to Conscientiousness, which is defined by behavior that is goal-oriented, organized, and responsible. Athletes from China who score high on the conscientiousness scale tend to be very organized when it comes to their training programs, putting in consistently good effort and never letting their guard down in a game. That quality makes athletes stick to the goal, work, and achieve their long-term goals and successes on the field. The capacity to overcome obstacles and maintain high performance is aided by the dependability and persistence that are associated with Conscientiousness. An athlete’s competitive spirit and capacity to perform under duress are bolstered by extraversion, which is characterized by sociability, aggressiveness, and excitement. Team setups and very pleasant surroundings, which are prevalent in many sports, tend to be ideal for extroverted athletes. Their positive attitude and boundless energy have the power to inspire others and raise morale on the team. Additionally, teams benefit from the leadership and communication skills of extroverted athletes, who often do better individually.

In team sports (Table 4), where cooperation and harmonious relationships are paramount, agreeableness—defined as a cooperative and sympathetic nature—is vital. Athletes with a high agreeableness score are more likely to work together as a team, resolve conflicts amicably, and cheer each other on. This quality is essential for a team’s success since it promotes cooperation, unity, and mutual respect. In addition, being amiable may improve the coach-athlete connection, which in turn improves communication and comprehension of training goals and methods. There is a multifaceted relationship between neuroticism and performance in athletics. The study results revealed that neuroticism is characterized by emotional instability and anxiety. Some athletes find that a moderate amount of neuroticism motivates them to strive for excellence and stay vigilant. The excessive degrees of neuroticism are often harmful owing to elevated stress and unpleasant emotional reactions. Consistent and resilient performance is a result of an athlete’s ability to handle competitive pressure, keep focused, and rebound from setbacks. Athletes with lower levels of neuroticism are more suited to do this. By developing physically coping mechanisms and engaging in regular mental training, athletes may lessen the impact of neuroticism and learn to channel their emotions positively. However, the Big Five character attributes have a positive impact on Chinese athletes’ performance by shaping their emotional resilience, social dynamics, flexibility, and diligence. All of these characteristics come together to form a profile that helps athletes perform at a high level. Having an open mind promotes creativity and flexibility; being disciplined and goal-oriented is the result of Conscientiousness; being extroverted makes one more competitive and better at interacting with others; being agreeable makes one more effective in teams and fosters cooperative dynamics; and, when controlled, neuroticism helps with emotional stability and motivation. All involved individuals, including athletes, coaches, and even sports psychologists, stand to gain a lot in a bid to enhance their understanding of personality qualities and their applicability in planning effective training schedules and strategies for exceeding expectations’ performance.

**Table 4:** TODIM method prediction as per fuzzy hierarchy alternatives (FAH).

FAH	*r*^*s*^_(i)_	$\Phi_{ik}^{j(-)}$	$G_{ik}^{j}$	$\mu_{\hat{d}}(x)$	*w*_*j*_
C11	1	0.613	(0.779, 0.248, 0.872)	0.753	2.774
C12	2	0.285	(0.677, 0.704, 0.649)	0.425	1.044
C13	3	0.152	(0.916, 0.108, 0.887)	0.518	1.236
C21	4	0.136	(0.916, 0.588, 0.221)	0.715	0.425
C22	5	0.264	(0.189, 0.168, 0.989)	0.241	0.274
C23	6	0.314	(0.261, 0.788, 0.323)	0.432	1.825
C31	7	0.699	(0.235, 0.961, 0.865)	0.135	0.145
C32	8	0.942	(0.794, 0.218, 0.819)	0.278	1.777
C33	9	0.158	(0.893, 0.161, 0.118)	0.565	6.675
**C1**	10	0.248	(0.567, 0.325, 0.675)	0.191	0.756
**C2**	11	0.243	(0.591, 0.254, 0.923)	0.642	2.332
**C3**	12	0.411	(0.228, 0.272, 0.499)	0.907	3.672

**Fig 3:**
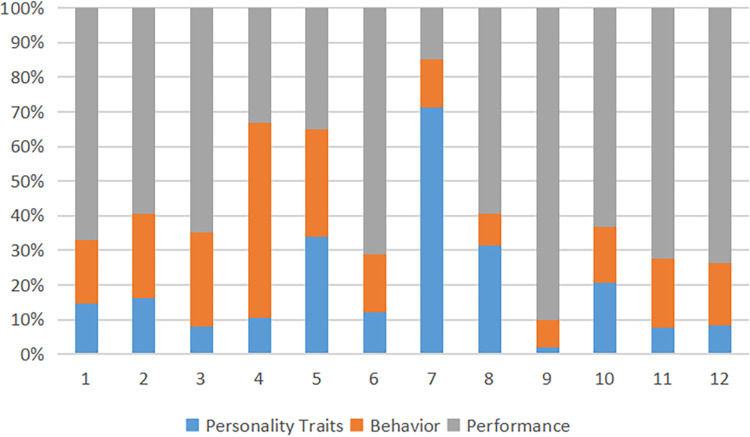
Fuzzy series matrix of study variables.

Participants from China shed more light on the effects of the Big Five factors on well-being and achievement, describing how these characteristics influence it (Fig 3). This study focuses on the analysis of participating Chinese athletes’ personality characteristics, such as Openness, Conscientiousness, extraversion, agreeableness, and neuroticism, and their impact on their behavior and performance at athletic events. Individuals relying on openness for their athletic performance profit from it essentially for the same reasons: strategic flexibility requires one to have the capacity to think unusually and uncommonly, which makes athletes develop uncompromised curiosity and enthusiasm for novelty. Those clients who scored higher on openness to experience and learn are rather inclined to try out new techniques, which may well increase performance levels. The characteristic feature of a growth mindset is the capacity to continue striving for progress when everything becomes difficult, which assists athletes. Discipline and traditional practices have been highly valued in Chinese sports, and therefore, even an athlete ready to adopt new things will find ways to do so and possibly increase their performance. The personality dimension that holds a lot of importance is Conscientiousness since it deals with dependability, orderliness, and goal orientation, thus ensuring success among the athletes. China’s performers in the conscientiousness dimension exhibit hard work, meticulous planning, and determination, which are characteristic of high achievements. This quality is particularly useful in organized sports, especially when particular procedures and training programs must be followed strictly. The probability of an athlete’s triumph rises when they are mindful of creating realistic goals, following through, being consistent with their training schedule, and engaging in deliberate practice (Table 5). Also, Chinese culture rewards perseverance, which can be traced to high Conscientiousness and ensures that the players focus on their activity.

**Table 5:** TODIM error detection fuzzy simulation analysis.

	β_1_			β_2_			β_3_		
	(1)	(2)	(3)	(1)	(2)	(3)	(1)	(2)	(3)
e_1_	0.845	0.957	0.785	0.197	0.564	0.263	0.566	0.286	0.915
e_2_	0.608	0.138	0.327	0.871	0.495	0.435	0.348	0.217	0.967
e_3_	0.527	0.131	0.874	0.966	0.728	0.548	0.302	0.259	0.236
e_4_	0.102	0.834	0.399	0.852	0.071	0.262	0.214	0.596	0.955
e_5_	0.504	0.173	0.549	0.281	0.577	0.791	0.646	0.788	0.972
e_6_	0.449	0.873	0.673	0.147	0.324	0.318	0.875	0.222	0.862
e_7_	0.155	0.948	0.901	0.525	0.111	0.588	0.943	0.434	0.149
e_8_	0.894	0.313	0.159	0.501	0.462	0.664	0.342	0.426	0.204
e_9_	0.894	0.758	0.875	0.882	0.101	0.103	0.507	0.731	0.935
e_10_	0.558	0.174	0.818	0.943	0.991	0.212	0.218	0.709	0.244
e_11_	0.306	0.595	0.165	0.892	0.618	0.219	0.299	0.365	0.532
e_12_	0.634	0.369	0.161	0.941	0.122	0.406	6.265	0.142	0.509

Extroverted athletes will perform better in team exercises and competitions because they are friendly, self-assured, and expressive. Perfectionism and assertiveness also strike highly extroverted Chinese athletes to do well in competitive situations or even when there are many spectators to watch. Players with higher levels of introversion are reported to engage in extroverted exercise, and the player’s interpersonal communication is critical when it comes to team-type sports. They are always happy and push people to work and achieve, so their presence is good and motivating. In addition, because of the high level of extroverted athletes the effects of increasing the performance as well as overall physical of the athletes are also manifested as a result of the efforts to seek social support or when solving various problems. In a nutshell, Turvill, Crust, and Langley established that athletes’ interpersonal relations could be predicted by their level of agreeableness, hence the extent to which the athletes preferred to empathize, collaborate, and trust one another. The agreeableness index entails the ability of Chinese athletes to play together, their ability to resolve conflicts, and their manners (Table 6). A synergy that involves common work with other people is highly helpful when it comes to team sports, where collaboration is the key to victory.

**Table 6:** Estimating risk bias of the fuzzy items in the TODIM method.

Variables	C11	C12	C13	C21	C22	C23	C31	C32	C33	Rank
C1	0.647	0.944	0.291	0.534	0.532	0.478	0.341	0.576	0.254	2
C2	0.597	0.693	0.718	0.948	0.549	0.404	0.044	0.548	0.341	3
C3	0.675	0.586	0.681	0.742	0.765	0.482	0.292	0.468	0.456	1

As much as the players may have their problems and may not agree on most aspects of life when they set their problems aside and work toward a particular aim, the team will do well. Athletes of individual sports ought to be agreeable, as this creates a supporting structure with coaches, other teammates, and mentors, improves the athletes’ experience, and improves their performance in the long run.

### 4.2 TODIM decision-making assessments

As for neuroticism, the situation is quite ambiguous: it is not only unrelated to athletic behavior in any particular way but also seems to possess an inverse correlation with athletic behavior as well. Neuroticism is, thus, rooted in hypersensitivity to and proneness to experience negative emotions. Thus, Chinese athletes with high levels of neuroticism could have problems with stress management, worrying, and uncertainty, all of which have a negative impact on performance. Nervosity, on the other hand, might lead the athletes to do everything possible and plan in such a way that they will avoid making a mistake. Sports culture in China could be a good context in which AI learns the skills of emotional regulation because Chinese practice focuses on mental strength. It is up to the coaches, the sports psychologists, in particular, alongside fellow athletes, to assist these characters in harnessing this energy within them and using it constructively.

Extending it, the elements of the Big Five personality have a major influence on behaviors concerning preparation, performance, and relationships among Chinese athletes. The personality traits of openness, Conscientiousness, extraversion, agreeableness, and neuroticism are as follows: While openness leads to creativity and flexibility, Conscientiousness is characterized regard to being precise and purposeful; sociability and assurance stem from extraversion; neuroticism, although it may be a drawback, can indeed prompt thorough preparation and endurance. It is important to note that all people are unique and thus require individualized psychological support and training programs, which factors these features into consideration for coaches, sports psychologists, and athletes. Promising athletic performance and physical outcomes for Chinese athletes can occur where opportunities for each of the identified attributes are maximized while difficulties of the other are minimized (Table 7).

**Table 7:** TODIM decision-making findings.

Time Horizon	Alternatives	Criteria	
		Athletic Behavior	Sports Performance
Baseline	A1.1	0.145	0.678
	A1.2	0.549	0.767
	A1.3	0.654	0.713
2008–2012	A1.1	0.717	0.913
	A1.2	0.593	0.119
	A1.3	0.017	0.568
2013–2018	A1.1	0.307	0.313
	A1.2	0.627	0.579
	A1.3	0.019	0.249
2019–2022	A1.1	0.698	0.002
	A1.2	0.187	0.053
	A1.3	0.172	0.973

Thus, recognizing how gender impacts the behavior and performance of Chinese athletes will help in using differences that male and female athletes have towards the activity in question. Thus, this research investigates gender differences to gain insight into how female and male athletes in China manage training, competitions, and relationships. Many of the typical sports that are considered male require and favor aggressiveness and high levels of competitiveness among male athletes. Especially when it comes to combat, in musically oriented quick-twitch sports, this spirit could be advantageous. Males further embark on risk-taking that puts them more at risk of getting an injury. At the same time, they are more at risk of achieving giant breakthroughs. Using data from the previous section on gender roles and expectations, it can also be noted that there is a tendency for male athletes to be the ones in charge or take up more responsibility in the team, which instills accountability and responsibility, hence, better performance and team cohesion. But, Chinese women’s sports teams can highly value teamwork and emotional stability. Perhaps these qualities would be highly useful in team sports, as they are dependent on the players’ communication and cooperation.

This is basically because female athletes are socially wiser, more understanding, and more organized in establishing a better organizational workforce (Table 8). On the other hand, the problem might be personalized; thus, it might be accompanied by prejudice and social expectations that would compromise a person’s self-esteem and overall efficiency. Still, Chinese women athletes exhibit high adaptability and perseverance, especially in technical and tactical-oriented sports, in which they seem to perform phenomenally well.

**Table 8:** Relative weights of decision-making criteria.

Time Horizon	Criteria	
	Athletic Behavior	Spots Performance
Baseline	0.209	0.212
2008–2012	0.444	0.487
2013–2018	0.567	0.599
2019–2022	0.619	0.627

### 4.3 Sensitivity analysis

This trend also applies to the mental part of athletes’ performance, as there are significant differences between men and women (Table 9). The male athlete’s experience might be damaging their mental physical and general well-being due to pressure to perform and be ’masculine’. Burnout and low performance are some of the worst things that can happen to a workforce, and methods like stress busting as well as mental toughness are needed. Female athletes learning how to balance their athletic and personal selves might benefit from psychological help with such issues as low self-esteem, distorted body image, and career and family conflicts. Gender also plays a part when it comes to the type of sporting discipline that Chinese athletes frequently participate in. Sporting events have for long been a preserve of the male gender, and there is a clear distinction between the type of sport that is associated with the male gender and one that is associated with the female gender, such as weight lifting and wrestling on one group and gymnastic and figure skating on the other. These linkages add to gender-specific behavior and performance outcomes since they establish training conditioning schedules, competition exposure, and the social support available to the athletes.

**Table 9:** The robustness analysis with ρ = 1,2,⋯,5.

	Alternative		
	A1.1	A1.2	A1.3
P = 1	0.495	0.927	0.514
P = 2	0.747	0.442	0.255
P = 3	0.473	0.195	0.679
P = 4	0.921	0.156	0.557
P = 5	0.227	0.442	0.483
C1	0.702	0.627	0.887
C2	0.005	0.497	0.244
C3	0.728	0.614	0.473
$\Phi_{ik}^{j(-)}$	0.401	0.595	0.423
$G_{ik}^{j}$	(0.883, 0.995, 0.584)	0.827, 0.659, 0.884	(0.793, 0.981, 0.869)
$\mu_{\hat{d}}(x)$	0.193	0.143	0.881
*w*_*j*_	0.416	0.746	0.881

Thus, one can state that the actions and outcomes of Chinese athletes depend much on the gender of the athlete. Due to noted possession of high-intensity training accessories, male athletes tend towards aggression and leadership. This paper will reveal the challenges that female athletes face in society but find it fit to inform the reader that they excel in technical and strategic activities during games and are able to display high emotional intelligence and, notably, a sense of teamwork. Every athlete should be given an equal and fair chance, irrespective of gender. This is why the battle for gender equity is crucial. Therefore, awareness of these gaps is crucial to the coaches, sports psychologists, and legislatures who are involved in one manner or another in the sporting world. This paper raises the importance of developing the methods accounting for the peculiarities of male and female athletes with reference to china.

## 5. Conclusion, implications, and future research

### 5.1 Conclusion

The purpose of this paper is to look into how the "big five" personality traits affect sports success in China. The evaluation was filled out by 260 Chinese players, both men and women, and the study got cross-sectional answers. Techniques for fuzzy analysis were used to look at the study’s data. Fuzzy TODIM evaluation and fuzzy decision analysis were used to draw conclusions. There were five psychological traits that were found to be significant drivers of sports behaviour. Neuroticism, on the other hand, was not found to be significant. The way players behave during games is also important for how well the game is played. Athletic behaviour plays a big part in the link between psychological traits and sports success as well. Behaviour and sports ability are also affected by gender in a big way. Chinese school districts and sports organisations should work to promote psychological training programmes that build mental toughness and help students do their best. Putting value on girls’ sports chances to support gender equality would help students do better in school and college sports. The study findings are robust in the Chinese context revealing the athletic performance differs gender-wise and to overcome such differences there is a need to work on sports burnout behavior and stress-taking activities. For this reason, policymakers need to take these kinds of steps to help people do well in school.

### 5.2 Practical implications

The behaviors and learning achievements of athletes in their personal as well as in their sports practices are strongly related to the existing policies in Ganzhou city of Jiangxi province, China. They may construct an environment that fosters individual change and the players’ performance enhancement by implementing appropriate measures and providing reliable assets. List of deeds that legislators have to perform In this case, there are several practical steps that lawmakers have to perform. The first thing they need to do is invest in programs that assist athletes in becoming complete persons by actually incorporating psychological training sessions in their day-to-day routine. This aspect of the program should focus on the mental, emotional, and psychosocial aspects as they are critical for enhanced performance. This way, sports psychologists and personality development specialists shall be able to attend to the needs of athletes who will receive attention that is appropriate for their needs. Secondly, the improvement of the sports environment should be a second priority to create a culture of pushing for sports.

The empowered authorities should consider the aspects of training environments promoting values like respect, cooperation, and sportsmanship. Some of the activities that could be included in the initiative could be contracts, meetings, and media campaigns that aim at bringing out the importance of good relations and ethical standards in sports. The personal attributes that work in the world of sports ball as regards immediate character and performance, might well be nurtured through an ethos of respect and collaboration. Third, there have to be trained coaches working with the athletes and great sports facilities available. Governments should hire trained experts and purchase cutting-edge training equipment for sports centers. Helping athletes realize their mental and physical potential requires cutting-edge training tools and techniques. Another way to improve the quality of training is to establish connections with international sports organizations. This will allow for the sharing of best practices and the exchange of expertise. Lastly, for all-around development, it is essential to establish educational programs that combine academics with sports. Schools and institutions should be encouraged by policymakers to provide athletes with flexible courses to accommodate their demanding schedules. In order to foster growth holistically, these programs should place equal emphasis on academic and athletic performance. Athletes may avoid financial stress and devote more time to training and study with the help of scholarships and other financial incentives.

### 5.3 Future research

Correspondingly, the present research suggests distinctive future research guidance for upcoming researchers to enhance the theory and practices. It includes,

- First, Flexible education program initiatives in China need to be researched to find how it enhance sports performance by balancing academic activities, athletes’ psychology, and sports performance.
- Second, the unattended aspects of female Chinese sports athletes need focused research to bring the latest insights about how sports opportunities shape gender equality in this perspective.
- Last, an experimental study and longitudinal research could effectively enhance the knowledge of how psychological training programs enhance the mental resilience and sports game performance of Chinese athletes.

## Supporting information

S1 File (PDF)

S2 File (DOCX)
