# Supplementary material for: Exploring performance of athletic individuals: Tying athletic behaviors and big-five personality traits with sports performance
Source: PLoS One. 2024 Dec 2;19(12):e0312850. doi: 10.1371/journal.pone.0312850 (PMC11611207; doi:10.1371/journal.pone.0312850)
Supplement: S1 File — (PDF) [file pone.0312850.s001.pdf]

赣南卫生健康职业学院科学研究伦理审查申请表

申请日期：　　2024.4.12　　　　　　　　编号(NO)：20240102

项目名称：探索运动员的表现：将运动行为和五大人格体质与运动表现联系起来			
项目负责人：黎琼		职称：副教授	
电话：　15970797986	电子信箱：daizhitong@126.com		
研究单位：赣南卫生健康职业学院			
合作研究单位：		合作单位负责人：	
联系电话：	传真：	邮编：	
研究者：黎琼	职称：副教授	研究者：	职称：
研究者：肖多	职称：副教授	研究者：	职称：
研究者：曾琼	职称：高职讲师	研究者：	职称：
研究时间：　2023年1月至2024年7月			
研究课题来源：☐政府　☐基金会　☐公司　☐国际组织　☑其他：中国体育运动员，包括篮球、足球、游泳等			
递交审查资料：<br>☐实验方案　☑知情同意书　☐其他资料______<br>包括：试验用品安全性资料、生产企业资质证明、试验用品提供者的资质证明			
审查结果（是否同意申请人的实验方案）	伦理审查委员会意见	☑同意　☐不同意　☐修改<br>该项目经学校伦理委员会审核，符合国家科技部国科发财字（2006）398号文件《关于善待实验动物的指导性意见》的相关规定，同意申报研究项目。	
经办人签章（Operator）：<br>黎琼	伦理审查委员会秘书签章（Signature of Ethics Committee Secretary）：<br>潘建萍	伦理审查委员会主任委员签章（Signature of Ethics Committee Director）：<br>梁海洋	

伦理审查委员会公章（Signature of Ethics Committee）:

[illegible]

2024.4.12.

赣南卫生健康职业学院伦理审查委员会
3607040953110

赣南卫生健康职业学院科学研究伦理审查意见表
（涉及人的生命科学和医学研究）

申请日期：　2024 年 4　月 12　日　　　　　　伦理编号(NO)：20240102

研究基本信息					
申报项目名称	探索运动员的表现：将运动行为和五大人格体质与运动表现联系起来				
申报项目来源	赣南卫生健康职业学院				
项目申报负责人	黎琼	联系电话	15970797986	承担系部	文化体育劳动部
合作研究单位：		合作研究单位负责人		联系电话	
电子信箱：	daizhitong@126.com	拟研究时间：	2023 年　1 月　25 日至　2024 年　7 月　1 日		
研究课题来源：□纵向　□横向　□院级　□研究者发起　□多中心合作　☑其他 这是一项横断面研究，主要数据来自中国的篮球、足球、游泳等体育运动员<br>项目来源名称：______________________					
涉及人的生物医学研究具体内容					

免疫疗法<br>☑不涉及	□干细胞（□自体 □异体） □CAR-T 细胞 □PD-1/PD-L1 □其他：
遗传学内容	☑不涉及 □涉及（具体内容：＿＿＿＿＿＿＿＿＿＿）
高风险的医疗新技术<br>☑不涉及	□利用粒子发生装置等大型仪器设备实施毁损式治疗技术<br>□放射性粒子植入治疗技术 □肿瘤热疗治疗技术 □肿瘤冷冻治疗技术<br>□人工智能辅助诊断治疗技术 □组织、细胞移植技术 □人工心脏植入技术<br>□瘤苗治疗技术 □同种器官移植技术 □变性手术<br>□中枢神经系统手术戒毒 □基因治疗技术 □克隆治疗技术<br>□立体定向手术治疗精神病技术 □异基因干细胞移植技术<br>□自体干细胞和免疫细胞治疗技术 □基因芯片诊断和治疗技术<br>□断骨增高手术治疗技术 □异种器官移植技术等<br>□其他＿＿＿＿＿＿＿＿

真实性申明：

以上内容由本人亲自填写，为本次研究课题的实际内容，本人承诺提交材料完全真实，不存在商业贿赂或其他不当利益关系，若因本人故意隐瞒研究内容，未按实际情况填写导致出现的任何伦理风险和不良事件，后果由本人自己承担。

项目申报负责人签名：[illegible]　　时间：2024年 4 月 12 日

系部意见：☑同意申报　　□不同意申报

项目研究涉及临床情况说明属实。

系部主任（签名）：[illegible]　　时间：2024年 4 月 12 日

伦理审查专家意见

☑同意 □不同意<br>□作必要修改后同意<br>修改意见：<br>专家 1 签名：[illegible]	☑同意 □不同意<br>□作必要修改后同意<br>修改意见：<br>专家 2 签名：[illegible]	☑同意 □不同意<br>□作必要修改后同意<br>修改意见：<br>专家 3 签名：[illegible]

[本申请表共 2 页，建议双面打印]

<table>
<tr><td>研究目的及试验过程</td><td colspan="5">本文没有涉及生物医学研究。
本研究旨在探讨大五人格特质与体育运动行为之间的相互作用。本研究对 260 名中国运动员(包括男性和女性)进行了问卷调查。应用模糊分析技术对研究结果进行检验。模糊 TODIM 评价，并应用模糊决策分析技术进行推理。结果表明，大五人格特征对运动行为有显著的预测作用;然而，神经质被发现是微不足道的。运动运动员运动行为的相互作用在运动比赛表现中也很重要。运动行为在人格特质与运动表现之间的关系中所起的作用也很重要。性别在行为和运动表现中也起着重要作用。中国的教育机构和体育行政部门应努力鼓励心理训练项目，以提高心理弹性的有效表现。重视女性参与体育运动的机会，塑造性别平等，将促进学校和大学的体育表现。因此，决策者需要采取这样的举措，对个人进行健康的学术培养。</td></tr>
<tr><td>研究方法</td><td colspan="5">☐干预性 ☑观察性（☐前瞻性 ☐回顾性 ☑现况性） ☐样本采集 ☐信息数据 ☑问卷</td></tr>
<tr><td>研究对象</td><td colspan="2">☑健康人 ☐患者（疾病：______）</td><td>样本量</td><td colspan="2">分析使用的实际回答 26</td></tr>
<tr><td>样本采集 类型</td><td colspan="2">☐血液 ☐组织 ☑其他：无需样品</td><td>是否出境</td><td colspan="2">☐是 ☑否</td></tr>
<tr><td>样本采集 来源</td><td colspan="2">☐生物样本库 ☐既往留存 ☐计划采集</td><td>国际合作</td><td colspan="2">☐是 ☑否</td></tr>
<tr><td>研究涉及医学技术的具体名称</td><td colspan="2">/</td><td>其中，药物/器械/制剂是否在国内上市</td><td colspan="2">☐是 ☐否 ☑ 不涉及</td></tr>
<tr><td>涉及医学技术在研究院内开展情况</td><td colspan="5">☐已在临床上开展的常规诊疗 ☐已在临床上开展但超出常规诊疗技术
临床科室审核意见：______ 审核人签名：______
☐已获医务科批准临床使用的新技术 ☐未获医务科批准临床使用的新技术
医务科审核意见：______ 审核人签名：______
☐其他______</td></tr>
<tr><td>医学技术经费说明</td><td colspan="5">☐常规诊疗收费 ☐非常规诊疗收费（☐患者免费 ☐患者自费）
★需由患者自费的理由说明:</td></tr>
<tr><td colspan="3">是否超出说明书规定剂量或方法用药/器械/制剂</td><td colspan="3">☐是 ☐否 ☑不涉及</td></tr>
<tr><td colspan="3">是否超出说明书规定适应症用药/器械/制剂</td><td colspan="3">☐是 ☐否 ☑不涉及</td></tr>
<tr><td colspan="3">是否有前期有效的临床研究/基础研究/动物实验支持</td><td colspan="3">☐是 ☑否</td></tr>
<tr><td>试验药品（器械）名称</td><td>/</td><td>是否赠药（器械） ☐是 ☑否</td><td colspan="2">是否采购目录内</td><td>☐是 ☑否</td></tr>
<tr><td>是否使用安慰剂</td><td>☐是 ☐否 ☑不涉及</td><td>是否有基础治疗</td><td colspan="3">☐是 ☐否 ☑不涉及</td></tr>
<tr><td colspan="6">✧ 无基础治疗且必须使用安慰剂的原因:</td></tr>
<tr><td>弱势群体
☑不涉及</td><td colspan="5">☐未成年人 ☐孕妇或胎儿 ☐晚期肿瘤/癌症患者 ☐精神障碍患者
☐无阅读能力（文盲，视力障碍，智力障碍，意识障碍等） ☐高龄老人
☐其他：______
★必须纳入的原因:</td></tr>
</table>
