# Supplementary material for: Exploring performance of athletic individuals: Tying athletic behaviors and big-five personality traits with sports performance
Source: PLoS One. 2024 Dec 2;19(12):e0312850. doi: 10.1371/journal.pone.0312850 (PMC11611207; doi:10.1371/journal.pone.0312850)
Supplement: S2 File — (DOCX) [file pone.0312850.s002.docx]

**Appendix (1) : Informed Consent Letter for Respondents**

| Dear Respondent,  We are conducting the research on the study topic *“Exploring performance of athletic individuals: Tying athletic behaviors and Big-five personality traits with sports performance”* In order to collect data, we are conducting a survey seeking views from people who are students at high school and colleges. For this, our research want your valuable responses. We shall be extremely grateful if you will kindly find time to complete this questionnaire. The questionnaire does not require you to give your name, or to mention any of your information. The information collected from the survey will be kept confidential and used only for the purpose of the aforesaid research. If for any reason, e.g. conflict of interest, you deem it necessary to withdraw your response after submitting it to me, it is my promise that your right to withdraw shall be fully respected. After the survey is complete and the results have been compiled, I will be happy to share the findings with you if you so desire.  Your cooperation will be greatly appreciated. It would also be a great help to the cause of research in our country.  Thank you.  Qiong Li |
| --- |

**Appendix (2) : Informed Consent Letter for Guardians/College Heads/Club Coaches**

| Dear Sir/Madam  We are conducting the research on the study topic *“Exploring performance of athletic individuals: Tying athletic behaviors and Big-five personality traits with sports performance”* In order to collect data, we are conducting a survey seeking views from your students who are sports player. For this, our research want your valuable permission. We shall be extremely grateful to get your approval. The purpose our research is to inquire how the personality traits and behaviors of sports players influence their sports performance on playground. Therefore, this letter serves the purpose of informed consent for you to bring the matter in your knowledge before approaching your students. The information collected from the survey will be kept confidential and used only for the purpose of the aforesaid research. If for any reason, e.g. conflict of interest, you deem it necessary to withdraw your response after submitting it to me, it is my promise that your right to withdraw shall be fully respected. After the survey is complete and the results have been compiled, I will be happy to share the findings with you if you so desire. Your cooperation will be greatly appreciated. It would also be a great help to the cause of research in our country.  Thank you.  Qiong Li |
| --- |
